# Actin maintains synaptic transmission by restraining vesicle release probability

**DOI:** 10.1016/j.isci.2025.112000

**Published:** 2025-02-14

**Authors:** Xin-Sheng Wu, Zhen Zhang, Yinghui Jin, Afreen Mushtaheed, Ling-Gang Wu

**Affiliations:** 1 National Institute of Neurological Disorders and Stroke, 35 Convent Dr., Bethesda, MD 20892, USA; 2 Office of Genetic Drugs, Center for Drug Evaluation and Research, Food and Drug Administration, 10903 New Hampshire Avenue, Silver Spring, MD 20993, USA

**Keywords:** Biological sciences, Neuroscience, Cell biology, Specialized functions of cells

## Abstract

Despite decades of pharmacological studies, how the ubiquitous cytoskeletal actin regulates synaptic transmission remains poorly understood. We addressed this issue with a tissue-specific knockout of actin β-isoform or γ-isoform, combined with recordings of postsynaptic EPSCs, presynaptic capacitance jumps or fluorescent synaptophysin-pHluorin changes, and electron microscopy in large calyx-type and small conventional hippocampal synapses. We found that actin restrains basal synaptic transmission during single action potential firings by lowering the readily releasable vesicle’s release probability. Such an inhibition of basal synaptic transmission is turned into facilitation during repetitive firings by slowing down depletion of the readily releasable vesicle pool and, thus, short-term synaptic depression, leading to more effective synaptic transmission for a longer time. These mechanisms, together with the previous finding that actin promotes vesicle replenishment to the readily releasable pool, may control synaptic transmission and short-term synaptic plasticity at many synapses, contributing to neurological disorders caused by actin cytoskeleton impairment.

## Highlights

• Actin β- and γ-isoform knockout impacts vesicle fusion and short-term depression
• Actin restrains readily releasable vesicle release probability
• Actin slows down short-term synaptic depression

## Graphical abstract

**Figure:**
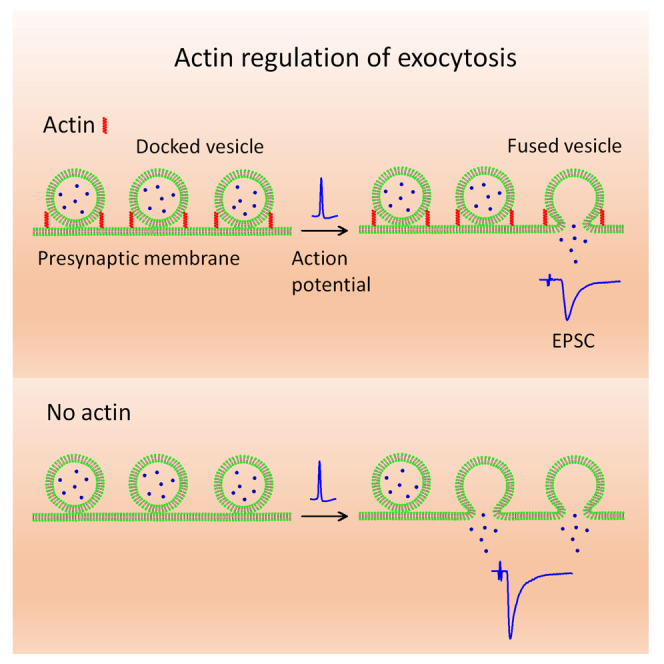

## Introduction

Being one of the most abundant cytoskeletal proteins, actin has been under intense scrutiny for decades regarding its role in synaptic transmission, often measured as the excitatory postsynaptic current (EPSC) or inhibitory postsynaptic current evoked by single presynaptic action potentials.^1^^,^^2^ However, these studies, with exclusive use of pharmacological tools to perturb filamentous actin (F-actin), yielded apparently controversial results. For example, latrunculin A or B, which inhibits F-actin formation by sequestering G-actin, increased the EPSC amplitude and the frequency of miniature EPSC (mEPSC) that reflects quantal release at hippocampal neurons,^3^ and increased vesicle release at inner hair cell ribbon synapses.^4^^,^^5^ In contrast, latrunculin does not enhance the EPSC or release at lamprey synapses,^6^ the EPSC at calyx-type synapses,^7^^,^^8^ or vesicle fusion at goldfish bipolar nerve terminals.^9^ Studies using cytochalasin D, another F-actin inhibitor, also showed contradictory results: cytochalasin D enhanced exocytosis at inner hair cell ribbon synapse^5^ but did not affect exocytosis at bipolar cell^10^ or the EPSC at hippocampal culture synapses where latrunculin A enhanced the EPSC.^3^ The reasons for these apparent controversies are unclear. Potential possibilities include (1) the use of pharmacological agents that disrupt the actin cytoskeleton to different degrees in different studies or induce off-target effects^11^ and (2) differential access to different actin pools in the same cell or different cell types.^6^ In brief, pharmacological studies regarding whether F-actin enhances or inhibits the EPSC evoked by single action potentials remain unresolved. How actin regulates basal synaptic transmission is thus unclear.

Instead of using pharmacological approaches, here we used conditional actin gene knockout approach to study whether and how actin regulates the EPSC at two synapses: (1) the calyx of Held synapses where presynaptic mechanisms can be directly detected with capacitance recordings,^12^^,^^13^ and (2) the cultured hippocampal synapse, a conventional synapse where pH-sensitive synaptophysin-pHluorin (SypH) imaging can be used to study presynaptic mechanisms.^14^^,^^15^ We provided the first evidence from the gene knockout approach suggesting that actin inhibits the EPSC by restricting the release probability of the readily releasable vesicles. We found that such an inhibitory role on basal synaptic transmission is converted into facilitation of synaptic transmission during repetitive action potential firing by slowing down the depletion of the readily releasable vesicles.

## Results

### β-actin knockout enhances the EPSC by increasing vesicle release

To determine whether actin regulates the EPSC, we knocked out β-cytoplasmic actin (β-actin) encoded by *Actb*, a dominant actin isoform in the brain,^16^ including the calyx of Held synapse.^17^ The knockout was tissue-specific because we bred *Actb*^*LoxP/LoxP*^ mice with *Krox20*^*Cre*^ mice, where Krox20-driven Cre activity is limited to the lower auditory system, including the calyx of Held synapse,^17^^,^^18^; subsequently, we bred *Krox20*^*Cre/+*^; *Actb*^*LoxP/+*^ mice with *Actb*^*LoxP/LoxP*^ mice, yielding *Krox20*^*Cre/+*^; *Actb*^*LoxP/LoxP*^ mice (termed here as *Actb*^*−/−*^ mice), in which β-actin was deleted in calyces, as demonstrated in detail previously.^17^

**Figure 1:**
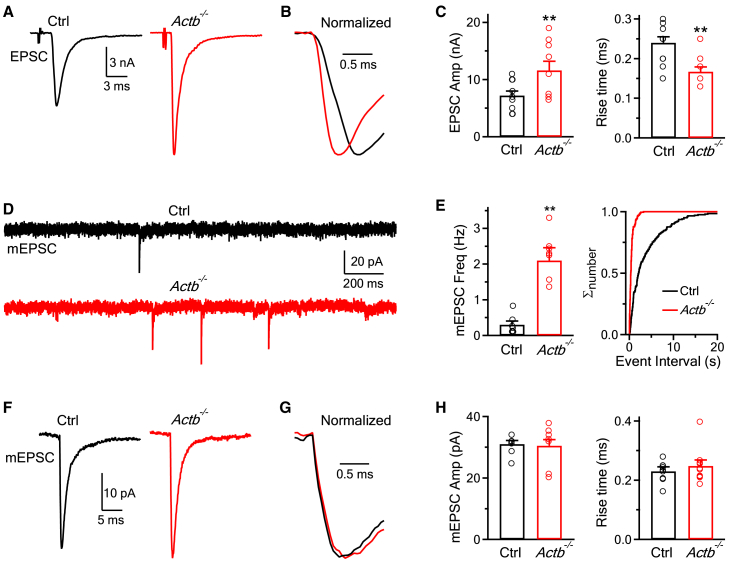
β-actin knockout increases the EPSC amplitude and the mEPSC frequency, but not the mEPSC amplitude at the calyx of Held synapse (A) Sampled EPSC evoked by a single presynaptic action potential in the control (Ctrl, left) or *Actb*^*−/−*^ (right) calyx of Held synapse. Vertical scale bar: 3 nA; horizontal scale bar: 3 ms. (B) Two EPSC traces in panel (A) are normalized to the same amplitude and superimposed for comparison of their rise time. Horizontal scale bar: 0.5 ms. (C) The EPSC amplitude (EPSC Amp, left) and 20–80% rise time (right) in Ctrl (10 neurons, from 5 male mice and 5 female mice) or *Actb*^*−/−*^ (9 neurons, from 5 male mice and 4 female mice) calyx of Held synapses. Each circle represents one neuron from a mouse. ∗∗: *p* < 0.01 (unpaired t test). (D) Sampled mEPSC traces from Ctrl (upper) or *Actb*^*−/−*^ (lower) calyx of Held synapses. Vertical scale bar: 20 pA; horizontal scale bar: 200 ms. (E) Left: the mEPSC frequency (mEPSC Freq, mean + SEM) in Ctrl (7 neurons, from 4 male mice and 3 female mice) or *Actb*^*−/−*^ (7 neurons, from 4 male mice and 3 female mice) calyx of Held synapses. Each circle represents one neuron from a mouse. ∗∗: *p* < 0.01 (unpaired t test). Right: cumulative number (normalized to 1, Σ_number_) plotted versus the inter-mEPSC interval from Ctrl (202 mEPSCs) or *Actb*^*−/−*^ synapses (389 mEPSCs). (F) Sampled mean mEPSC trace from a Ctrl (left, averaged from 30 mEPSCs of a neuron) or a *Actb*^*−/−*^ calyx of Held synapse (right, averaged from 69 mEPSCs of a neuron). Vertical scale bar: 10 pA; horizontal scale bar: 5 ms. (G) Two mEPSC traces in panel (F) are normalized to the same amplitude and superimposed for comparison of their rise time. Horizontal scale bar: 0.5 ms. (H) The mEPSC amplitude (mEPSC Amp, left, mean + SEM) and 20–80% rise time (right, mean + SEM) of Ctrl (7 neurons, 7 neurons, from 4 male mice and 3 female mice) and *Actb*^*−/−*^ (7 neurons, 7 neurons, from 4 male mice and 3 female mice) synapses. Each circle represents one neuron from a mouse.

To determine whether the EPSC is affected in *Actb*^*−/−*^ mice, we prepared transverse brainstem slices (200 μm thick) containing the medial nucleus of the trapezoid body (MNTB) from 7 to 10 days old male or female mice.^19^^,^^20^ A bipolar stimulating electrode was positioned at the midline of the trapezoid body where presynaptic axon fibers pass through. A stimulus (5–20 V, 0.1 ms) was delivered via the stimulating electrode every 20 s to induce a presynaptic action potential. The resulting EPSC was recorded with a whole-cell voltage clamp of the postsynaptic principal neurons in the MNTB at room temperature (22‒24°C, Figure 1A).^19^^,^^20^

In control mice (see STAR Methods for control mice), the EPSC amplitude was 7.2 ± 0.8 nA (10 calyces, 10 mice), and the 20–80% rise time of the EPSC was 0.24 ± 0.02 ms (10 neurons, 10 mice, Figures 1A–1C). In *Actb*^*−/−*^ mice, the EPSC amplitude was 12.7 ± 1.6 nA (9 neurons, 9 mice), which was ∼76% (=12.7/7.2-1) larger than that (7.2 ± 0.8 nA) in control mice (t test, *p* = 0.005; Figures 1A–1C); the 20–80% rise time of the EPSC was 0.17 ± 0.01 ms (9 neurons, 9 *Actb*^*−/−*^ mice), which was significantly shorter than that (0.24 ± 0.02 ms) of control mice (t test, *p* = 0.004, Figures 1A–1C).

To determine whether β-actin knockout affects the quantal response, we recorded the miniature excitatory postsynaptic current (mEPSC). The mEPSC frequency in *Actb*^*−/−*^ mice (2.1 ± 0.36 Hz, 7 neurons, 7 mice) was significantly higher than that (0.30 ± 0.10 Hz, 7 neurons, 7 mice) in control mice (t test, *p* = 0.0004, Figures 1D and 1E); whereas the mEPSC amplitude and rise time were similar between *Actb*^*−/−*^ and control mice (Figures 1F–1H). Thus, β-actin knockout increased the EPSC amplitude, the EPSC rise speed, and the mEPSC frequency, but not the mEPSC amplitude or rise speed, implying that β-actin knockout increases the number of vesicles being released by single action potentials at the calyx.

### β-actin or γ-actin knockout increases the release probability of readily releasable vesicles at calyces

Postsynaptic current recordings described previously imply enhanced release in *Actb*^*−/−*^ nerve terminals. To confirm this implication, we performed whole-cell voltage-clamp recordings at the calyx of Held^12^^,^^13^ to directly determine whether β-actin knockout (or γ-actin knockout) enhances release and the mechanisms involved. The underlying mechanisms may encompass an increased release probability of the readily releasable vesicles (Prob_RRV_), a larger readily releasable vesicle pool (RRP) size, and/or an increased calcium current (ICa).

**Figure 2:**
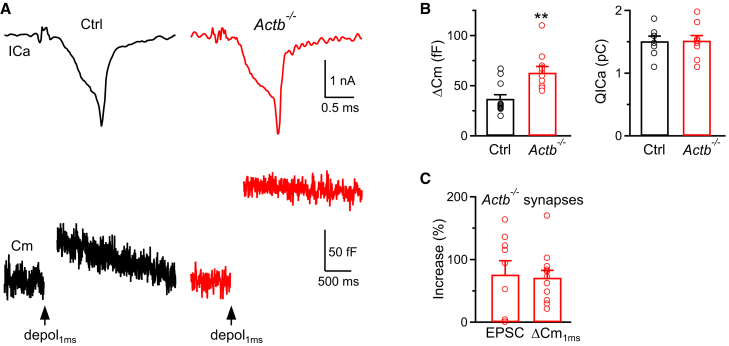
β-actin knockout increases the EPSC by increasing vesicle fusion at calyces (A) Sampled Ca^2+^ current (ICa, upper) and membrane capacitance (Cm, lower) jump (ΔCm) induced by 1 ms depolarization (depol_1ms_) from −80 mV to +10 mV in a Ctrl (left) or *Actb*^*−/−*^ (right) calyx. The first 250 ms Cm traces after depolarization are not shown owing to potential artifact contamination. ICa and Cm traces are shown in different timescales. Upper vertical scale bar: 1 nA; upper horizontal scale bar: 0.5 ms. Lower vertical scale bar: 50 fF; lower horizontal scale bar: 500 ms. (B) ΔCm (mean + SEM) and ICa charges (QICa, mean + SEM) induced by 1 ms depolarizations in Ctrl (13 calyces, from 6 male mice and 7 female mice) or *Actb*^*−/−*^ (12 calyces, from 6 male mice and 6 female mice) calyces. ∗∗: *p* < 0.01 (unpaired t test). Each circle represents one neuron from a mouse. (C) Normalized ΔCm (mean + SEM, 12 calyces, from 6 male mice and 6 female mice) induced by 1 ms depolarizations and the normalized EPSC (mean + SEM, 9 neurons, from 5 male mice and 4 female mice) induced by 1 AP from *Actb*^*−/−*^ synapses are plotted together to show their similar increases in *Actb*^*−/−*^ synapses. Data (ΔCm from Figure 2B; EPSC from Figure 1C) were normalized to their corresponding mean value in the Ctrl group. Each circle represents one neuron from a mouse.

At the whole-cell voltage-clamp configuration with pipette and bath solutions that isolated ICa (see STAR Methods),^12^^,^^13^ we applied a 1-ms depolarization from the holding potential of −80 mV to +10 mV to mimic an action potential.^13^^,^^21^ This 1-ms depolarization induced a calcium current (ICa), and a capacitance jump (ΔCm, Figures 2A and 2B).^13^^,^^21^ The ΔCm induced by 1-ms depolarization (ΔCm_1ms_) increased from 37 ± 4 fF in control (13 calyces, 13 mice) to 63 ± 6 fF in *Actb*^*−/−*^ calyces (12 calyces, 12 mice, t test, *p* = 0.003) (Figures 2A–2C). This reflected a ∼70% (=63/37-1) increase of the mean ΔCm_1ms_, similar to the ∼76% increase of the EPSC observed in *Actb*^*−/−*^ mice (Figure 2C). Similar percentage increases in ΔCm_1ms_ and the EPSC (Figure 2C) suggest that β-actin knockout increases the EPSC by enhancing exocytosis.

**Figure 3:**
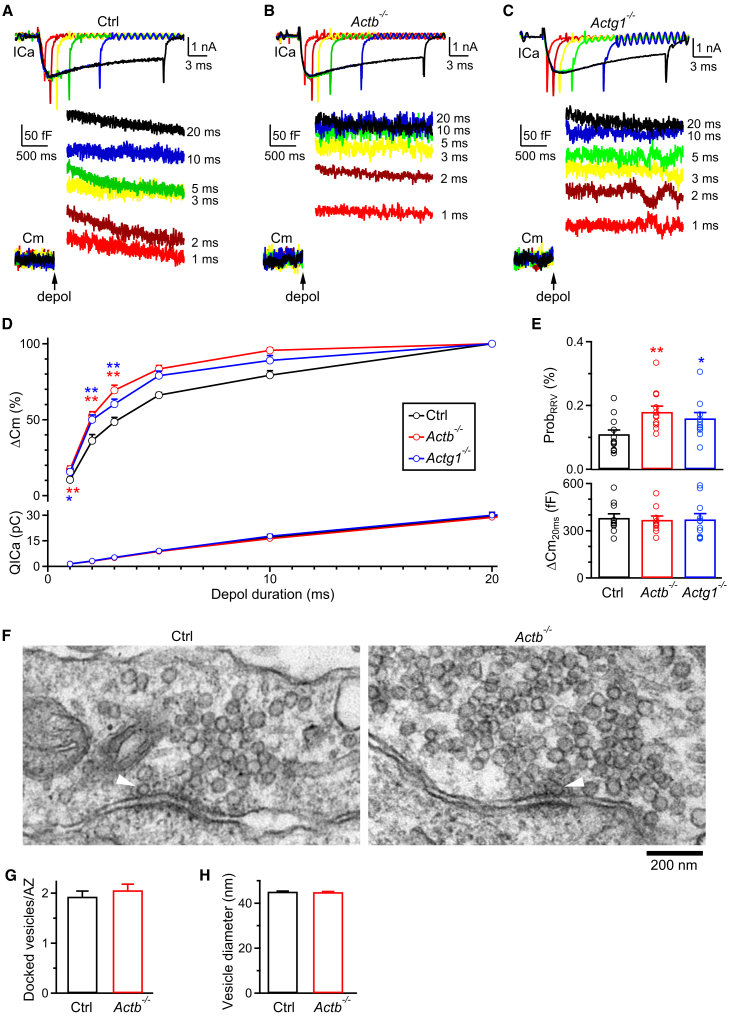
β- and γ-actin knockout increase vesicle fusion by increasing the release probability of readily releasable vesicles at calyces (A) Sampled Ca^2+^ current (ICa, upper) and membrane capacitance (Cm, lower) jump (ΔCm) induced by 1 (red), 2 (brown), 3 (yellow), 5 (green), 10 (blue), or 20 (black) ms depolarizations from −80 mV to +10 mV in a Ctrl calyx. The arrow indicates the onset of depolarization (depol). The first 250 ms Cm traces after depolarization are not shown owing to potential artifact contamination. ICa and Cm traces are shown in different timescales. The interval between each depolarization is ∼30–60 s. Upper vertical scale bar: 1 nA; upper horizontal scale bar: 3 ms. Lower vertical scale bar: 50 fF; lower horizontal scale bar: 500 ms. (B and C) Similar to panel (A), but with the Ctrl calyx replaced by an *Actb*^*−/−*^ calyx (B) or by an *Actg1*^*−/−*^ calyx (C). (D) ΔCm (mean + SEM) and ICa charges (QICa, mean + SEM) induced by 1, 2, 3, 5, 10, or 20 ms depolarizations (depol) in Ctrl (black, 13 calyces, from 6 male mice and 7 female mice), *Actb*^*−/−*^ (red, 12 calyces, from 6 male mice and 6 female mice) and *Actg1*^*−/−*^ (blue, 11 calyces, from 5 male mice and 6 female mice) calyces. ΔCm data are normalized to ΔCm evoked by the 20 ms depol in the same calyx. ∗: *p* < 0.05 (unpaired t test) and ∗∗: *p* < 0.01 (unpaired t test) for comparison between Ctrl and *Actb*^*−/−*^ group (red) or between Ctrl and *Actg1*^*−/−*^ group (blue) at 1, 2, and 3 ms depol. (E) Prob_RRV_ (mean + SEM, upper) and ΔCm (lower) induced by the 20 ms depol (ΔCm_20ms_, reflecting the RRP size) in Ctrl (black, 13 calyces, from 6 male mice and 7 female mice), *Actb*^*−/−*^ (red, 12 calyces, from 6 male mice and 6 female mice) and *Actg1*^*−/−*^ (blue 11 calyces, from 5 male mice and 6 female mice) calyces. Prob_RRV_ = ΔCm_1ms_/ΔCm_20ms_. Each circle represents one calyx from a mouse. (F) Sampled electron micrographs of a part of a Ctrl (left) or *Actb*^*−/−*^ (right) calyx. Arrowheads indicate docked vesicles: vesicles physically touch the dense active zone membrane. Horizontal scale bar: 200 nm. (G) The number (mean + SEM) of docked vesicles per active zone (AZ) in Ctrl (147 AZs from 2 male mice and 2 female mice) or *Actb*^*−/−*^ (139 AZs from 2 male mice and 2 female mice) calyces. (H) Vesicle diameter (mean + SEM) in Ctrl (619 vesicles, 20 calyx cross sections from 2 male mice and 2 female mice) or *Actb*^*−/−*^ (628 vesicles, 23 calyx cross sections from 2 male mice and 2 female mice) calyces.

ICa charge (QICa) induced by the 1-ms depolarization was similar between control and *Actb*^*−/−*^ calyces (Figures 2A and 2B), suggesting that the enhanced release is caused by a mechanism downstream of calcium influx. To determine whether the enhanced release is due to increased Prob_RRV_ or RRP, we applied 1, 2, 3, 5, 10, or 20 ms depolarizations from the holding potential of −80 mV to +10 mV every 30 s (Figures 3A–3D). A 20-ms depolarization depleted the RRP,^12^^,^^13^^,^^20^^,^^21^^,^^22^ whereas a 1–5 ms depolarization partially depleted the RRP (Figures 3A–3D). The capacitance jump (ΔCm) induced by the 20-ms depolarization (ΔCm_20ms_) reflected the RRP size; the ratio between ΔCm_1ms_ and ΔCm_20ms_ (ΔCm_1ms_/ΔCm_20ms_) reflected the Prob_RRV_ in response to an action potential-equivalent stimulus.^12^^,^^13^^,^^20^^,^^21^^,^^22^

ΔCm_20ms_ was similar between control and *Actb*^*−/−*^ calyces, suggesting that β-actin knockout does not affect the RRP size (control mice: 381 ± 26 fF, *n* = 13 calyces from 13 mice; *Actb*^*−/−*^ mice: 375 ± 23 fF, *n* = 12 calyces from 12 mice; t test, *p* = 0.86) (e.g., Figures 3A and 3B; summarized in Figure 3E). In contrast, ΔCm_1ms_/ΔCm_20ms_ in *Actb*^*−/−*^ calyces was significantly higher than in control calyces (Figures 3A, 3B, 3D, and 3E), suggesting that β-actin knockout substantially enhances the Prob_RRV_.

Consistent with the increase of ΔCm_1ms_/ΔCm_20ms_, the ΔCm_2ms_/ΔCm_20ms_, and ΔCm_3ms_/ΔCm_20ms_ in *Actb*^*−/−*^ calyces were also significantly higher than those in control calyces, respectively (Figure 3D). The ICa charges induced by 1–20 ms depolarization in *Actb*^*−/−*^ calyces were similar to those in control calyces, respectively, suggesting that β-actin knockout enhances the Prob_RRV_ by mechanisms downstream of calcium influx.

To determine whether the increased Prob_RRV_ in *Actb*^*−/−*^ calyces is specific to only the β-isoform, we also generated *Krox20*^*Cre/+*^; *Actg1*^*LoxP/LoxP*^ mice (termed *Actg1*^*−/−*^ mice) by breeding *Krox20*^*Cre/+*^; *Actg1*^*LoxP/+*^ mice with *Actg1*^*LoxP/LoxP*^ mice (see STAR Methods).^17^ Capacitance recordings in calyces showed that analogous to β-actin knockout, γ-actin knockout (*n* = 11 calyces from 11 mice) increased the ΔCm_1ms_, ΔCm_1ms_/ΔCm_20ms_, but not ΔCm_20ms_ or the QICa (Figures 3C–3E), suggesting that like β-actin knockout, γ-actin knockout also enhances the Prob_RRV_.

### Electron microscopy reveals no change in the docked vesicles

Capacitance measurements revealed no change in the RRP size in *Actb*^*−/−*^ calyces (Figures 3A–3E). Here, we performed electron microscopy (EM) to examine if morphologically docked vesicles are changed in *Actb*^*−/−*^ calyces. Docked vesicles were counted as those in direct contact with the dense presynaptic membrane, the active zone membrane (Figure 3F).^23^^,^^24^ The number of docked vesicles per active zone in a cross-section was similar between control and *Actb*^*−/−*^ calyces; the vesicle diameter was also comparable (Figures 3F–3H). These results were consistent with the similar RRP size detected with ΔCm_20ms_ in control and *Actb*^*−/−*^ calyces (Figures 3A–3E).

### β-actin knockout speeds up short-term depression of synaptic transmission

**Figure 4:**
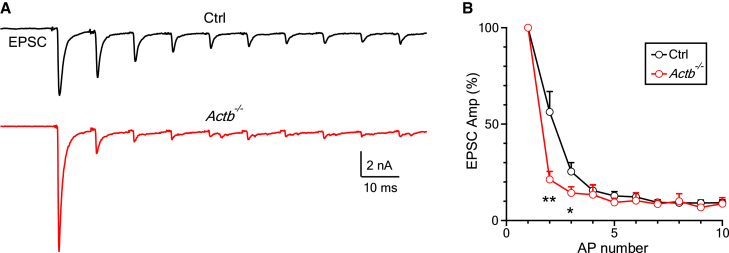
β-actin knockout speeds up short-term depression of the EPSC during repetitive firing at the calyx of Held synapse (A) Sampled EPSC traces induced by 10 APs at 100 Hz in a Ctrl (upper) or *Actb*^*−/−*^ (lower) calyx of Held synapse. Vertical scale bar: 2 nA; horizontal scale bar: 10 ms. (B) The amplitude of the EPSC (EPSC Amp, mean + SEM) evoked by each of the 10 presynaptic APs at 100 Hz in Ctrl (9 neurons, from 5 male mice and 4 female mice) or *Actb*^*−/−*^ (9 neurons, from 5 male mice and 4 female mice) calyx of Held synapses. The EPSC Amp was normalized to the first EPSC. ∗∗: *p* < 0.01 at AP number 2; ∗: *p* < 0.05 at AP number 3 (unpaired t test).

We found that β-actin knockout increased basal synaptic transmission (evoked by single action potentials) by enhancing Prob_RRV_ (Figures 1, 2, and 3). To determine whether β-actin knockout affects synaptic transmission during repetitive firing, we recorded EPSCs induced by 10 action potentials (AP) applied at 100 Hz via the bipolar stimulating electrode at the midline of the trapezoid body. The EPSC amplitude decreased rapidly during the stimulation train, reaching ∼10% of the EPSC evoked by the 1^st^ AP in both *Actb*^*−/−*^ and control mice (Figures 4A and 4B), which reflected short-term depression of transmitter release.^20^^,^^21^^,^^22^^,^^25^ The amplitude of the EPSC evoked the 2^nd^ and the 3^rd^ AP of the train decreased by a more significant percentage in *Actb*^*−/−*^ mice (21 ± 4% and 14 ± 3% of the 1^st^ EPSC, 9 calyces, 9 mice) than in control mice (56 ± 11% and 25 ± 5%, of the 1^st^ EPSC, 9 calyces, 9 mice), revealing a faster short-term depression in *Actb*^*−/−*^ mice (Figures 4A and 4B). Since the enhanced release evoked by the 1^st^ AP caused more depletion of the RRP, a more depleted RRP may account for the larger depression observed during the 2^nd^ and 3^rd^ AP of the train.

### β-actin knockout increases the release probability at hippocampal synapses

**Figure 5:**
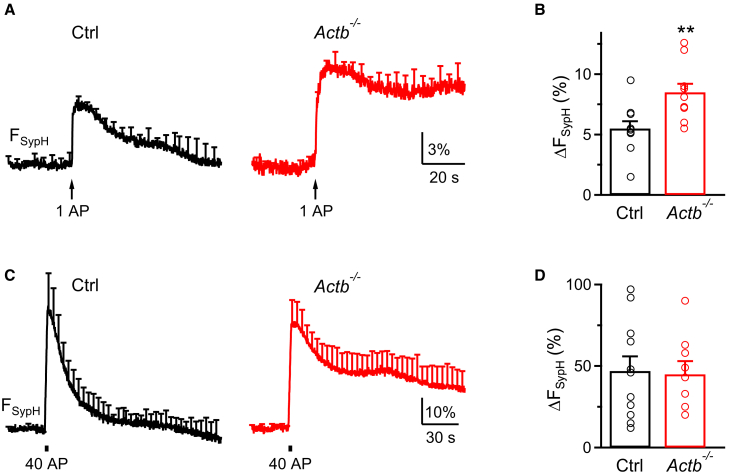
β-actin knockout enhances vesicle exocytosis at hippocampal neurons (A) SypH fluorescence (F_SypH_, mean + SEM) changes induced by 1 AP (arrow) in Ctrl (left, 10 experiments) or *Actb*^*−/−*^ (right, 10 experiments) hippocampal boutons. SEM is plotted every 4 s. Each experiment contained 20–30 boutons from 1 culture; each culture was from 3 to 5 mice (applies to A–D). Vertical scale bar: 3%; horizontal scale bar: 20 s. (B) ΔF_SypH_ (mean + SEM) induced by 1 AP in Ctrl (10 experiments) or *Actb*^*−/−*^ hippocampal boutons (10 experiments). Each circle denotes ΔF_SypH_ from each experiment. ∗∗: *p* < 0.01, unpaired t test. (C and D) Similar to (A) and (B), respectively, except that 1 AP is replaced with 40 AP at 20 Hz (bar). Ctrl, 11 experiments; *Actb*^*−/−*^, 9 experiments. Vertical scale bar in (C): 10%; horizontal scale bar in (C): 30 s.

We found that β- or γ-actin knockout increases the Prob_RRV_ at the large calyx of Held synapses (Figures 1, 2, and 3). To determine whether this result applies to small conventional synapses, we examined exocytosis at hippocampal synapses cultured from *Actb*^*LoxP/LoxP*^ mice. Cultured *Actb*^*LoxP/LoxP*^ neurons were transfected with SypH (synaptophysin tagged with the pH-sensitive pHluorin2X)^26^ either alone as control or with a plasmid containing Cre-mCherry for ≥7 days to generate *Actb*^*−/−*^ boutons in culture (see ref.^17^ for detail). In control synapses, an action potential (AP) induced SypH fluorescence increase (ΔF_SypH_) to 5.5 ± 0.6% (normalized to the baseline, 10 experiments), whereas 40 APs at 20 Hz, which depletes the RRP,^27^^,^^28^^,^^29^ induced a ΔF_SypH_ of 47 ± 9% (11 experiments). The ratio between ΔF_SypH_ induced by 1 AP and that (the RRP size) by 40 AP may thus reflect the RRP vesicle release probability during an AP.^27^^,^^28^^,^^29^ In *Actb*^*−/−*^ boutons, ΔF_SypH_ induced by 1 AP was 8.5 ± 0.7% (10 experiments), significantly larger than the control (t test, *p* = 0.004; Figures 5A and 5B), whereas ΔF_SypH_ induced by 40 APs was 45 ± 8% (9 experiments), similar to the control (t test, *p* = 0.41; Figures 5C and 5D). These results suggest that β-actin knockout enhanced exocytosis induced by 1 AP by increasing the RRP vesicle release probability at hippocampal synapses (Figure 5), similar to the results obtained in calyx-type synapses (Figures 1, 2, and 3).

Although the ΔF_SypH_ induced by an AP, in principle, reflects the balance of exocytosis and endocytosis plus vesicular reacidification, the endocytosis plus reacidification should be insignificant during an AP that lasts for ∼1–2 ms, because a 1–2 ms endocytosis plus reacidification have not been reported, to our knowledge.^30^^,^^31^^,^^32^ Furthermore, ΔF_SypH_ induced by an AP was similar in the presence or absence of bafilomycin that blocks vesicle reacidification (Figure S1), confirming that ΔF_SypH_ induced by an AP reflects exocytosis. The ΔF_SypH_ increase in *Actb*^*−/−*^ boutons may thus reflect the increase of exocytosis.

We showed previously that β-actin knockout reduced ΔF_SypH_ induced by 200 APs at 20 Hz, suggesting the involvement of actin in replenishing the RRP.^17^ This suggestion was further verified in the calyx of Held, where β-actin knockout reduced the rate of the RRP replenishment as measured with capacitance recordings.^17^ In the present work, we also verified the previous observation that ΔF_SypH_ induced by 200 APs was larger than that induced by 40 APs at the same cultures (Figure S2), and was inhibited by β-actin knockout. Thus, while restricting the Prob_RRV_, β-actin enhances the RRP replenishment as reported previously.^17^

## Discussion

We studied the roles of actin in synaptic transmission in large calyx-type and small hippocampal synapses using the knockout approach for the first time, combined with measurements of the postsynaptic EPSCs and mEPSCs, presynaptic ΔCm, SypH imaging, and EM. In large calyx-type synapses, β-actin knockout enhanced the basal EPSC evoked by single APs (Figure 1) and ΔCm_1ms_ (release similar to an AP, Figure 2) to a similar percentage but did not affect the mEPSC amplitude (Figure 1) or presynaptic ICa (Figures 2 and 3), suggesting a presynaptic mechanism enhancing the EPSC. This enhancement is not due to the RRP size increase but the Prob_RRV_ increase, as detected in both *Actb*^*−/−*^ and *Actg1*^*−/−*^ calyces (Figure 3), suggesting that both β- and γ-actin are involved in restraining the Prob_RRV_. β-actin knockout enhanced Prob_RRV_ in both the calyx of Held and hippocampal boutons (Figure 5). While enhancing basal synaptic transmission, β-actin knockout speeded up short-term depression during the repetitive AP train (Figure 4), suggesting that the enhanced Prob_RRV_ by the β-actin knockout depleted more RRP vesicles during the repetitive AP train, leading to faster short-term depression. Taken together, our results suggest that cytoskeletal protein actin restrains the RRP vesicle release probability at nerve terminals but facilitates synaptic transmission during repetitive stimulation by slowing down short-term depression. This conclusion might apply to most mammalian central synapses because the supporting data were obtained from both the large calyx-type synapse and the conventional small bouton synapse in the mammalian central nervous system.

In the last two decades, pharmacological studies regarding whether F-actin enhances or inhibits the EPSC evoked by single action potentials have been controversial (see introduction). With the knockout approach, our results suggest that actin plays dual roles: an inhibitory role for basal synaptic transmission by reducing the Prob_RRV_ but a facilitatory role during repetitive nerve firing by slowing down the RRP depletion. These findings, together with the previous finding that actin facilitates the RRP replenishment,^7^^,^^8^^,^^17^^,^^33^ suggest that actin facilitates synaptic transmission during repetitive firing by both reducing the Prob_RRV_ to slow down RRP depletion and by facilitating the RRP replenishment via active zone clearance after exocytosis.^17^^,^^33^ Although actin plays both inhibitory and facilitatory roles, the inhibitory role in basal synaptic transmission underlies the more important facilitatory role during repetitive firing, as physiological stimulations often appear as a train of repetitive AP trains, such as during the auditory processing of sounds.^34^ These mechanisms may be impaired by defects in the actin cytoskeleton signaling pathway, contributing to the generation of neurological disorders. For example, a mutation in the potassium channel Kv3.3 that causes spinocerebellar ataxia disrupts F-actin nucleation beneath the membrane, including nerve terminals.^35^^,^^36^ The impairment of F-actin nucleation in nerve terminals may enhance basal synaptic transmission but inhibit synaptic transmission during repetitive firing, causing faster short-term depression, which may contribute to the generation of spinocerebellar ataxia.

The Prob_RRV_ is controlled by many factors, such as the SNARE proteins, the calcium sensor synaptotagmin, proteins (e.g., Mover) associated with active zone scaffold proteins,^37^ and calcium channel numbers and positions at the active zone.^38^^,^^39^ The present work suggests updating this view (reviewed in the study by Korber C. and Kuner T.^38^) by including actin as a key molecule to restrain the Prob_RRV_. How does actin restrain the Prob_RRV_? Since dynamin 1 knockout or dynamin 1 and 3 double knockout inhibits endocytosis but does not affect or reduce the EPSC evoked by an AP,^40^^,^^41^^,^^42^ the increased Prob_RRV_ in *Actb*^*−/−*^ or *Actg1*^*−/−*^ nerve terminals is unlikely to be caused by developmental compensation to the inhibition of endocytosis previously reported in *Actb*^*−/−*^ or *Actg1*^*−/−*^ nerve terminals.^17^ It has been suggested that cortical actin beneath the plasma membrane is a physical barrier preventing vesicles from docking at the release site or being adjacent to calcium channels.^5^^,^^43^^,^^44^^,^^45^ However, this hypothesis predicts increased RRP size and replenishment but not the RRP vesicle release probability after actin disruption, which is different from what we observed. Depolymerization of actin may cause rearrangement of the active zone matrix,^46^; actin has been suggested to co-localize with a core exocytosis protein SNAP-25 in chromaffin cells^47^; annexin A2-induced actin bundling may regulate release site formation^45^; actin is densely present at the calyx of Held nerve terminal.^17^ These observations suggest that actin may contribute to forming the cytomatrix of the release site or the active zone to interact with the docking vesicle and the fusion machinery, resulting in restraining the RRP vesicle release. Many proteins, such as Mover, a protein that interacts with the active zone scaffolding protein bassoon, and VAMP4, a noncanonical vesicular SNARE, have been shown to affect the Prob_RRP_.^37^^,^^48^ It is possible that actin may interact with these Prob_RRP_-controlling proteins to restrain the Prob_RRP_. In addition, cortical actin beneath the plasma membrane maintains the membrane tension^49^^,^^50^^,^^51^ and may pull the plasma membrane inward to form an invagination.^17^^,^^52^^,^^53^^,^^54^ These forces might contribute to restricting RRP vesicle release. It would be of great interest in the future to find out the exact mechanism by which actin restrains vesicle release.^3^

### Limitations of the study

Our suggestion that actin maintains synaptic transmission by restraining vesicle release probability is based on conventional electrophysiological and imaging experiments that does not provide the molecular mechanism. Direct evidence of the mechanism with high temporal- and spatial-resolution light imaging is a future direction and is ultimately needed to solidify the main suggestion of the present work and to provide underlying molecular mechanisms.

## Resource availability

### Lead contact

Further information and requests for resources and reagents should be directed to and will be fulfilled by the lead contact Ling-Gang Wu (wul@ninds.nih.gov).

### Materials availability

This study did not generate new unique reagents.

### Data and code availability

All data produced for this manuscript are available from the lead contact (wul@ninds.nih.gov) upon reasonable request. This paper does not report original code. Any additional information required to reanalyze the data reported in this paper is available from the corresponding author upon request.

## Acknowledgments

This work was supported by the National Institute of Neurological Disorders and Stroke Intramural Research Program in United States (ZIA NS003009-15 and ZIA NS003105-10 to L.-G.W.). We thank Dr. James M. Ervasti (University of Minnesota, Minneapolis, USA) and Patrick Charnay (10.13039/501100009517PSL Research University Paris, France) for providing *Actb*^*LoxP/LoxP*^, *Actg1*^*LoxP/LoxP*^, and *Krox20*^*Cre*^ mice, respectively. We thank Dr. Yongling Zhu (Northwestern University, Chicago) for providing synaptophysin-pHluorin2X plasmid.

## Author contributions

X.-S.W. and L.-G.W. designed the experiments and supervised the project. X.-S.W., Z.Z., and Y.J. performed experiments and analyzed the data with help from A.M. L.-G.W. wrote the paper with help from all authors.

## Declaration of interests

The authors declare no competing interests.

## STAR★Methods

### Key resources table

**Table:** 

REAGENT or RESOURCE	SOURCE	IDENTIFIER
**Chemicals, peptides, and recombinant proteins**		

German glass coverslips with mouse laminin coating over PDL layer	Neuvitro	Cat#GG-25-Laminin
Horseradish peroxidase (HRP)	Sigma-Aldrich	Cat#P8125
Lipofectamine LTX with PLUS	Thermo Fisher Scientific	Cat#15338030
Papain	Worthington	Cat#LK003178
Tetrodotoxin (TTX)	Tocris	Cat#1078

**Critical commercial assays**		

Elite ABC-HRP kit	Vector Labs	Cat#PK-6100
Quick extract DNA extraction solution	Lucigen	Cat#QE09050

**Experimental models: Organisms/strains**		

*Actb*^*LoxP/LoxP*^ mice	James M. Ervasti lab	N/A
*Actg1*^*LoxP/LoxP*^ mice	James M. Ervasti lab	N/A
*Krox20*^*Cre*^ mice	Patrick Charnay lab	N/A

**Recombinant DNA**		

pH-sensitive pHluorin 2X (SypH)	Yong-Ling Zhu lab	N/A

**Software and algorithms**		

Clampfit 9 (for Axopatch 200B amplifier)	Molecular Devices	https://www.moleculardevices.com
Igor Pro 6.1.2.1	WaveMetrics, Inc.	https://www.wavemetrics.com
Image J	National Institutes of Health	https://imagej.nih.gov/ij
NIS-Elements AR 4.1 (for Nikon Eclipse Ti microscope)	Nikon	https://www.microscope.healthcare.nikon.com
Pulse v8.67 (for EPC-10 amplifier)	HEKA Elektronik	https://www.heka.com

**Others**		

Axopatch 200B amplifier	Molecular Devices	https://moleculardevices.com
EPC-10 Amplifier	HEKA Elektronik	https://www.heka.com
Integraslice 7550 Vibratome	Campden Instruments	https://www.campdeninstruments.com
iXon X3 897 CCD digital camera (for SypH imagine)	Andor Technology	https://andor.oxinst.com
JEM-200 CX transmission electron microscope	JEOL	https://www.jeolusa.com
Nikon Eclipse Ti microscope	Nikon	https://www.microscope.healthcare.nikon.com
XR-100 CCD digital camera (for EM imagine)	AMT	https://amtimaging.com

### Experimental model and study participant details

#### Animals

Animal care and use were carried out according to NIH guidelines and were approved by the NIH Animal Care and Use Committee (NINDS ASP 1170).

As described previously,^17^ we generated tissue-specific β-actin (*Actb* gene) knockout (KO) mice by crossing *Actb*^*LoxP/LoxP*^ mice with *Krox20*^*Cre*^. *Actb*^*LoxP/LoxP*^ mice, originally provided by Dr. James M. Ervasti (University of Minnesota, Minneapolis, MN),^55^^,^^56^ were obtained by homozygous breeding using standard mouse husbandry procedures. *Krox20*^*Cre*^ mice were provided by Dr. Patrick Charnay (PSL Research University, Paris, France).^57^ We crossed *Krox20*^*Cre/+*^ mice with *Actb*^*LoxP/LoxP*^ mice and obtained the expected 50% Cre-positive and *Actb*^*LoxP/+*^ offspring. By further crossing *Krox20*^*Cre/+*^;*Actb*^*LoxP/+*^ mouse line with *Actb*^*LoxP/LoxP*^ mouse line, we got the expected 25% *Krox20*^*Cre/+*^;*Actb*^*LoxP/LoxP*^ offspring mice (termed *Actb*^*-/-*^ mice), which were identified with PCR-based genotyping and used for experiments. The Cre-negative *Actb*^*LoxP/LoxP*^ littermates were used as control mice. Immunostaining showed the presence of β-actin at the calyx in control mice, but not in *Actb*^*-/-*^ mice, indicating successful removal of β-actin in the calyx of *Actb*^*-/-*^ mice.^17^ Similar to the generation of *Actb*^*-/-*^ mice, we generated tissue-specific γ-actin (*Actg1* gene) knockout (KO) mice by crossing *Actg1*^*LoxP/LoxP*^ mice with *Krox20*^*Cre*^ (for detail, see ref.^17^).

P7-10 mice of either sex were used for the preparation of brainstem slices. P0 mice of either sex were used for hippocampal culture experiments. The present work used mice of either sex randomly.

#### Primary cell cultures

Hippocampal CA1-CA3 region from P0 male or female mice was dissected, dissociated, and plated on Poly-D-lysine treated coverslips.^14^^,^^29^

### Method details

#### Brain slice preparation and recordings of mEPSCs, EPSCs and membrane capacitance

Transverse brainstem slices (200 μm thick) containing the medial nucleus of the trapezoid body (MNTB) were prepared from 7−10 days old male and female mice using a vibratome in ice-cold cutting solution containing (in mM): 125 NaCl, 2.5 KCl, 3 MgCl_2_, 0.1 CaCl_2_, 25 NaHCO_3_, 1.25 NaH_2_PO_4_, 25 glucose, 0.4 ascorbic acid, 3 *myo*-inositol, 2 sodium pyruvate, pH 7.4 when bubbled with 95% O_2_ and 5% CO_2_.^12^^,^^58^ Slices were incubated in the bath solution for 30 min at 37°C and then held at 22 – 24°C. Bath solution contains (in mM): 125 NaCl, 2.5 KCl, 1 MgCl_2_, 2 CaCl_2_, 25 NaHCO_3_, 1.25 NaH_2_PO_4_, 25 glucose, 0.4 ascorbic acid, 3 *myo*-inositol, 2 sodium pyruvate, pH 7.4 when bubbled with 95% O_2_ and 5% CO_2_. All experiments were carried out at 22 – 24°C.

For postsynaptic whole-cell voltage-clamp recordings of EPSCs and mEPSCs in transverse slices, an Axopatch 200B amplifier was used. Postsynaptic pipette (2 – 3 MΩ) solution contained (in mM): 125 K-gluconate, 20 KCl, 4 MgATP, 10 Na_2_-phosphocreatine, 0.3 GTP, 10 HEPES, 0.5 EGTA, pH 7.2, adjusted with KOH. The series resistance (< 15 MΩ) was compensated by 95% (lag 10 μs). The bath solution contained (in mM): 125 NaCl, 2.5 KCl, 1 MgCl_2_, 2 CaCl_2_, 25 NaHCO_3_, 1.25 NaH_2_PO_4_, 25 glucose, 0.4 ascorbic acid, 3 *myo*-inositol, 2 sodium pyruvate, 0.05 D, L-2-amino-5-phosphonovaleric acid (AP-5), 0.01 bicuculline and 0.01 strychnine, pH 7.4 when bubbled with 95% O_2_ and 5% CO_2_. A bipolar stimulating electrode was positioned at the midline of the trapezoid body, through which the presynaptic axons of MNTB synapses pass. A stimulus (5–20 V, 0.1 ms) was delivered every 20 s to induce a presynaptic action potential, and the resulting EPSC was recorded at the postsynaptic cell.^59^ The postsynaptic neurons of the MNTB were whole-cell voltage-clamped at a holding potential of –80 mV, a potential at which AMPA receptors mediate the EPSC.

Whole-cell capacitance measurements of the calyces at parasagittal slices were made with the EPC-10 amplifier with a software lock-in amplifier (1000 Hz sine wave, peak-to-peak voltage ≤ 60 mV). We pharmacologically isolated presynaptic Ca^2+^ current with a recording solution (∼22–24°C) containing (in mM): 105 NaCl, 20 TEA-Cl, 2.5 KCl, 1 MgCl_2_, 2 CaCl_2_, 25 NaHCO_3_, 1.25 NaH_2_PO_4_, 25 glucose, 0.4 ascorbic acid, 3 *myo*-inositol, 2 sodium pyruvate, 0.001 TTX, 0.1 3,4-diaminopyridine, pH 7.4 when bubbled with 95% O_2_ and 5% CO_2_. The presynaptic pipette contained (in mM): 125 Cs-gluconate, 20 CsCl, 4 MgATP, 10 Na_2_-phosphocreatine, 0.3 GTP, 10 HEPES, 0.05 BAPTA, 0.05 AP-5, pH 7.2, adjusted with CsOH. Membrane capacitance was measured within 10 min after break-in to avoid rundown.^58^ For stimulation, we used a depolarization with various durations (1, 2, 3, 5, 10, and 20 ms ) from the holding potential of −80 mV to +10 mv. A 20 ms depolarization can deplete the vesicles in the RRP. The first 250 ms of capacitance recordings after depolarization were not used (and thus not shown in figures) to avoid capacitance artifact contamination.^60^^,^^61^

#### Electron microscopy at the MNTB

P8-9 male and female mice were anesthetized deeply with Nembutal (80 mg/kg body weight) via intraperitoneal route and transcardially perfused with 4% paraformaldehyde. The brains were removed and stored overnight in fresh fixative at 4°C. 100-μm-thick slices containing MNTB complex were cut using a vibratome. Slices were washed with 0.1 N cacodylate buffer before trimming. Samples after trimming were treated with 1% OsO_4_ in cacodylate buffer for 1 hour on ice, and 0.25% uranyl acetate in acetate buffer at pH 5.0 overnight at 4°C, dehydrated with ethanol series, and embedded in epoxy resin. Thin sections were counterstained with 2% uranyl acetate and lead citrate. Serial ultrathin sections (∼90 nm in thickness) from epoxy resin embedded specimens were cut with a diamond knife (Diatome) in an ultramicrotome UC7 (Leica Microsystems) and mounted onto mesh grids. Sections were counterstained with lead citrate for 5 min.

Digital micrographs of ultra-thin sections through the MNTB were acquired at 120 kV in JEOL JEM-200cx electron microscope equipped with a bottom-mounted CCD digital camera system at a primary magnification of 20,000X and analyzed with ImageJ.

Measurement of vesicle diameter (d) is based on the equation: d = [(a1∗b1∗c1)^1/3^ + (a2∗b2∗c2)^1/3^]/2, where a1 = outer leaflet long axe, b1 = outer leaflet short axe, c1 = (a1 + b1)/2, and a2 = inner leaflet long axe, b2 = inner leaflet short axe, c2= (a2+ b2)/2.^62^ To estimate the distance between vesicles and the active zone membrane, the cross section must contain the dense active zone membrane. The vesicles that appeared in direct physical contact with the active zone membrane were considered docked vesicles.

#### Hippocampal neuron culture, imaging, and analysis

*Actb*^*LoxP/LoxP*^ mouse hippocampal culture was prepared as described previously.^14^^,^^29^^,^^63^ Briefly, hippocampal CA1-CA3 regions from P0‒P1 mice were dissected, dissociated, and plated on Poly-D-lysine treated coverslips. The sex of each P0‒P1 mouse was not determined; however multiple replicates should represent both males and females. Cells were maintained at 37°C in a 5% CO_2_ humidified incubator with a culture medium consisting of Neurobasal A, 1% GlutaMAX-1, 10% fetal bovine serum, 2% B-27, and 3 μM cytosine β-D-arabinofuranoside. On 5–7 days after plating, neurons were transfected with plasmids using Lipofectamine LTX.

*Actb*^*LoxP/LoxP*^ hippocampal cultures were transfected with the plasmids containing SypH alone (control) or with L309 plasmid containing Cre-mCherry. A nuclear localization sequence was tagged at the N-terminal of Cre, which was then cloned into L309 vector (a gift from Dr. Thomas Sudhof and Zhiping Pang, Stanford University) via BamHI and EcoRI sites. Imaging was performed at ≥ 7 days after transfection, at which Cre-mCherry transfection effectively generated *Actb*^*-/-*^ boutons.^17^ The deletion of β-actin was detected with western blotting (see Figures 6A–6C in ref.^17^).

The action potential was evoked by a 1 ms pulse (20 mA) through platinum electrodes. The bath solution contained (in mM): 119 NaCl, 2.5 KCl, 2 CaCl_2_, 2 MgCl_2_, 25 HEPES, 30 glucose, 0.01 6-cyano-7-nitroquinoxaline-2, 3-dione (CNQX), and 0.05 D, L-2-amino-5-phosphonovaleric acid, pH 7.4, adjusted with 1 M NaOH. In experiments, we heated the culture chamber using a temperature controller. Imaging was performed after the culture was at 34–37°C for 15–30 min. The temperature was verified with another small thermometer in the chamber. SypH images were acquired at 10 Hz using Nikon A1 confocal microscope (60X, 1.4 NA), and analyzed with Nikon software. All boutons showing SypH fluorescence (F_SypH_) increases (ΔF_SypH_) were analyzed (region of interest: 2 μm X 2 μm). ΔF_SypH_ was normalized to the baseline F_SypH_ before stimulation (baseline F_SypH_ was normalized as 100%). Such a normalization removed the impact of different experiments with different SypH expression levels. Each data group was obtained from at least three batches of cultures.

### Quantification and statistical analysis

#### Data collection and quantifications

EPSCs in brain slices were induced by pulses at 0.05 Hz to avoid the depression and potentiation.

Calyx capacitance was measured within 10 min after break-in to avoid rundown.^58^ The first 0.25 s trace after stimulation was not used (and thus not shown in figures) to avoid capacitance artifact contamination.^60^^,^^61^

For SypH signal in hippocampal cultures, F_SypH_ was normalized to the baseline F_SypH_ before stimulation (baseline F_SypH_ was normalized as 100%). Such a normalization removed the impact of different cultures on the measurement of ΔF_SypH_ after stimulation.

#### Statistical tests

Data were expressed as mean ± s.e.m. Replicates are indicated in results and figure legends. The statistical test used is *t* test. An asterisk (∗) indicates *p* < 0.05 (unpaired t test); two asterisks (∗∗) indicates *p* < 0.01 (unpaired t test). Although the statistics were performed based on the number of cells or experiments, each group of data was collected from at least four mice. For imaging in hippocampal cultures, each group of data was collected from at least 6 experiments; each experiment contained 20-30 boutons; 1-3 experiments were taken from 1 culture; each culture was from 3-5 mice.
