# Supplementary material for: Actin maintains synaptic transmission by restraining vesicle release probability
Source: iScience. 2025 Feb 14;28(3):112000. doi: 10.1016/j.isci.2025.112000 (PMC11919605; doi:10.1016/j.isci.2025.112000)
Supplement: Document S1. Figures S1 and S2 [file mmc1.pdf]

iScience, Volume *28*

Supplemental information

Actin maintains synaptic transmission by restraining vesicle release probability

Xin-Sheng Wu, Zhen Zhang, Yinghui Jin, Afreen Mushtaheed, and Ling-Gang Wu

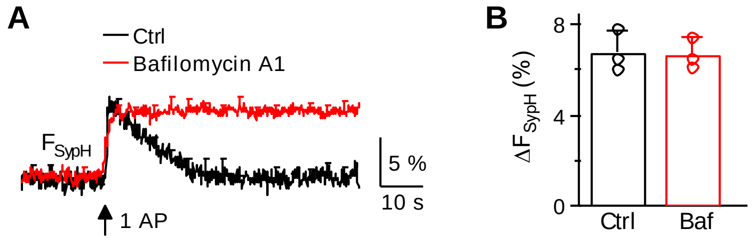

Figure S1. Bafilomycin does not affect the ΔF_{SypH} induced by 1 AP at hippocampal neurons, related to Figure 5.

(A) SypH fluorescence (F_{SypH}, mean + s.e.m.) changes induced by 1 AP (arrow) at hippocampal boutons in the absence (Ctrl, black trace, 3 experiments) or presence of 0.1 µM bafilomycin A1 (red trace, 3 experiments). F_{SypH} was collected after 20 min application of bafilomycin A1. s.e.m. is plotted every 4 s. Bafilomycin blocks vesicle reacidification, explaining why F_{SypH} did not decay after the jump induced by 1 AP. Vertical scale bar: 5%; horizontal scale bar: 10 s.

(B) F_{SypH} jump (ΔF_{SypH}, mean + s.e.m.) induced by 1 AP at hippocampal boutons in the absence (Ctrl, black, 3 experiments) or in the presence of 0.1 µM bafilomycin A1(Baf, red, 3 experiments). Each circle denotes ΔF_{SypH} from each experiment. ΔF_{SypH} was similar between two groups ($p > 0.05$, unpaired *t* test), indicating that ΔF_{SypH} induced by an AP reflects exocytosis.

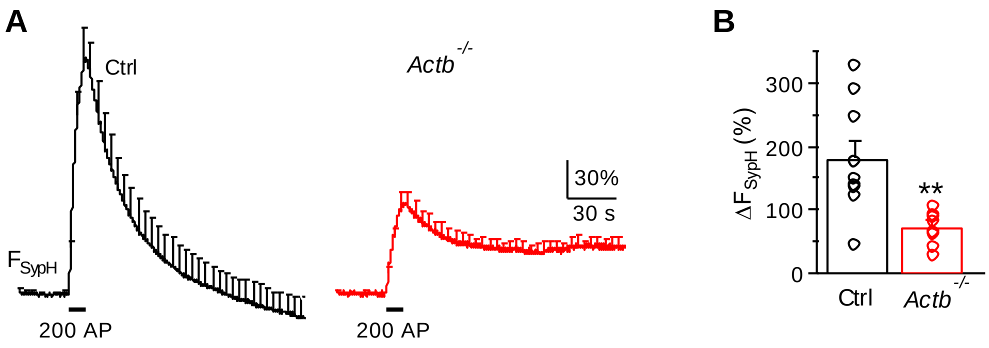

Figure S2. β-actin knockout decreases ΔF_{SypH} induced by 200 APs at hippocampal neurons, related to Figure 5.

(A) SypH fluorescence (F_{SypH}, mean + s.e.m.) changes induced by 200 AP at 20 Hz (bar) in Ctrl (left, 10 experiments) or *Actb*$^{-/-}$ (right, 8 experiments) hippocampal boutons. s.e.m. is plotted every 4 s. Scale bar applies to two graphs. Vertical scale bar: 30%; horizontal scale bar: 30 s.

(B) F_{SypH} jump (ΔF_{SypH}, mean + s.e.m.) induced by 200 AP at 20 Hz in Ctrl (10 experiments) or *Actb*$^{-/-}$ hippocampal boutons (8 experiments). Each circle denotes ΔF_{SypH} from each experiment. β-actin knockout decreases ΔF_{SypH} induced by 200 AP at 20 Hz (**: $p < 0.01$, unpaired *t* test), indicating that β-actin enhances the replenishment of the readily releasable pool of synaptic vesicles.
